# The Effect of Diet Composition on the Digestibility and Fecal Excretion of Phosphorus in Horses: A Potential Risk of P Leaching?

**DOI:** 10.3390/ani10010140

**Published:** 2020-01-15

**Authors:** Markku Saastamoinen, Susanna Särkijärvi, Elisa Valtonen

**Affiliations:** 1 Production Systems, Natural Resources Institute Finland (Luke), FI-31600 Jokioinen, Finland; susanna.särkijärvi@luke.fi; 2 Department of Animal Science, University of Helsinki, FI-00790 Helsinki, Finland; elisa.mj.valtonen@gmail.com

**Keywords:** environment, horse nutrition, phosphorus loss, phosphorus supplementation, phosphorus retention

## Abstract

**Simple Summary:**

This study aimed to examine phosphorus utilization and excretion in feces when typical feeds and forage-based diets are fed. The hypothesis was that feeding regimes might influence phosphorus digestibility and excretion in feces, and therefore the environmental impact of horse husbandry. We also studied the nutrient digestibilities of the diets, as well as the proportion of the soluble fraction of P of the total phosphorus. Horse dung may pose a potential risk of P run-off into the environment if not properly managed. Supplementation with inorganic P should be controlled in the diets of mature horses in light work to decrease the excretion of P in feces.

**Abstract:**

The main horse phosphorus excretion pathway is through the dung. Phosphorus originating from animal dung and manure has harmful environmental effects on waters. The number of horses has increased in many countries, and several studies have pointed that leaching of P from horse paddocks and pastures are hotspots for high P leaching losses. The hypothesis was that feeding regimes might influence phosphorus digestibility and excretion in feces, and therefore the environmental impact of horse husbandry. A digestibility experiment was conducted with six horses fed six forage-based diets to study phosphorus utilization and excretion in feces. The study method was a total collection of feces. The experimental design was arranged as an unbalanced 6 × 4 Latin Squares. Phosphorus intake increased with an increasing concentrate intake. All studied diets resulted in a positive P balance and, the P retention differed from zero in all except the only-hay diet, in which the intake was lower compared to the other diets. The digestibility of P varied from 2.7 to 11.1%, and supplementing forage-diets with concentrates slightly improved P digestibility (*p* = 0.024), as it also improved the digestibilities of crude protein (*p* = 0.002) and organic matter (*p* = 0.077). The horses excreted an average of 20.9 ± 1.4 g/d P in feces. Excretion was smallest (20.0 g) in horses on a hay-only diet (*p* = 0.021). The average daily phosphorus excretion resulted in 7.6 kg P per year. The soluble P part of the total P in feces accounted for about 88% of the P excreted in feces, and is vulnerable to runoff losses and may leach into waters. Thus, horse dung may pose a potential risk of P leaching into the environment if not properly managed, and is not less harmful to the environment than that from other farm animals. Supplementation with inorganic P should be controlled in the diets of mature horses in light work to decrease the excretion of P in feces.

## 1. Introduction

Macro-mineral phosphorus plays an important role in bone formation as a constituent of phosphoproteins, phospholipids, and nucleic acids, and in energy and fat metabolism [1,2,3]. In animals, 80–85% of phosphorus is stored in the bones and teeth, and the remainder in soft tissues and body fluids [3]. In the skeleton, phosphorus forms hydroxyapatite with calcium. In growing animals, the need for phosphorus is greater than in adult animals, since developing bones require more phosphorus than already developed bones in adult animals [4].

Phosphorus deficiency is found throughout the world in areas with soil poor in phosphorus. Deficiency symptoms include, but are not limited to, rice disease, osteomalacia, nervous system symptoms, stiff joints, muscle weakness, poor fertility, impaired ovarian function and consequent irregular rotation, poor growth in juvenile animals, and impaired weight gain in adult animals [1]. To avoid these detrimental effects of phosphorus malnutrition and ensure efficient intake, phosphorus is usually routinely supplemented in horses’ diets.

Phosphorus absorption is influenced by the intake, source, and composition of the feed ration [5]. In adult horses, which mainly eat roughage, absorption efficiency is 35%, and in lactating mares and growing horses it is 45% as their diets are often supplemented by larger amounts of concentrates [1]. There may be some improvement in phosphorus absorption as the need for phosphorus increases, for example, through exercise or when the phosphorus content of the diet increases [6]. Fowler et al. [7] concluded that yearlings can utilize organic P as well as mature horses.

The main site of the gastrointestinal tract of phosphorus absorption in horses is the dorsal colon, but some phosphorus is also absorbed from the small intestine [8,9]. Fowler et al. [7] suggested that degradation either occurs after the site of P absorption, or liberated P is recycled back into the gastrointestinal tract.

The main phosphorus excretion pathway is through the dung. The phosphorus content of feces is directly proportional to the phosphorus content of the diet [10]. Especially in a diet rich in forage, the horse often gets too much supplemented phosphorus to meet its needs, and excess P is excreted from the body in the feces [11]. A very low proportion (about 1%) of the phosphorus is excreted in the urine [8,10,12], and is thus usually ignored in studies dealing with P digestibility. In the gastrointestinal tract, endogenous phosphorus secretion occurs in the small intestine and in the cecum [8,13]. Endogenous phosphorus secretion is due to the presence of phosphorus compounds in gastrointestinal fluids such as saliva and gastric, pancreatic, and biliary fluids [8,14].

The digestibility of phosphorus is influenced by its form and amount in feed, and its interaction with other feed components and minerals, e.g., Ca and Ca:P ratio [1,15]. Cereals are good sources of phosphorus [3], but a significant proportion of the phosphorus in cereals is bound to phytic acid, which is poorly digestible in monogastric animals [16]. However, the horse is able to digest phytate phosphorus [7,17,18]. The content of phosphorus in grasses approximately equates to that in cereals (about 3 g/kg DM), but the phosphorus content of grasses is significantly influenced by the age of the crop at the time of harvest [19,20]. Mineral supplements usually contain inorganic forms of phosphorus like monocalcium phosphate, dicalcium phosphate, or phosphorite as phosphorus sources [4].

Phosphorus and nitrogen originating from animal manure are the main environmental and water pollutants from agriculture. There is imbalance of N and P in the manure, and P is considered more harmful, because when it is in excess of crop requirements, soil becomes saturated resulting in P runoff [21]. Because the number of horses has increased in many countries, and horses are kept in paddocks and pastures there is risk of P leaching to the environment also from horse husbandry. In addition, horse manure is widely utilized in agriculture. However, the NRC [1] considers horse feces to be less risky to the environment compared to that from other farm animals because it assumes that horse manure contains less water-soluble phosphorus, prone to runoff. However, later studies have pointed that leaching of P from horse paddocks and pastures are hotspots for high P leaching losses [22,23,24,25].

In general, dietary strategies have been developed for many animal species to effectively reduce the total P concentration in manure. As we were interested in the possible detrimental environmental impact of horses, we studied phosphorus utilization and excretion in feces applying typical feeds and forage-based diets fulfilling the current P -intake recommendations [1,19]. We also studied the nutrient digestibilities of the diets, as well as the proportion of the soluble fraction of P of the total phosphorus. The hypothesis was that feeding regimes might influence phosphorus digestibility and excretion in feces, and therefore the environmental impact of horse husbandry.

## 2. Materials and Methods 

A digestibility experiment was conducted with six forage-based diets typically fed to horses in Finland. The study was conducted at the facilities of the Natural Resources Institute Finland (Luke) in Southwest Finland. In animal handling and sample collection, the European Union recommendation directives (2010/63/EU) and national animal welfare and ethical legislation set by the Ministry of Agriculture and Forestry of Finland were followed carefully. The experimental procedures were evaluated and approved by the national ethical committee for animal experiments (https://www.avi.fi/web/avi/elainkoelautakunta-ella) (ESAVI/8331/04.10.07/2013).

### 2.1. Horses and their Management

Six adult Finnhorse mares (5–13 years; initial BW 552 ± 32 kg, mean BCS 6 = moderately fleshy) owned by Luke were used in the study. All the experimental horses had the same managing and feeding history before the trial. The horses were individually housed in stalls (3 m × 3 m) with peat as bedding. The horses were de-wormed before the experiment. Dental care and vaccinations had been carried out regularly prior to the experiment. The horses were freely exercised daily in groups in outdoor paddocks (with sand grounds) for 2–4 h, except during the collection period, when they were led in walk by a rope in the stable corridors (consisting of two connected 32 m long corridors with concrete and asphalt floors) for 15 min. In the paddocks, the horses had masks to prevent sand eating.

The study method was a total collection of feces. The experimental design was arranged as an unbalanced 6 × 4 Latin Squares. The study consisted of six treatments and four 21-day periods. Each period started with a five-day feed change period followed by 12 days of adaptation to the new diet, and four-day period of collecting feces samples. During the collection period, the peat bedding was changed to rubber mats. The body weight (BW) (electronic animal scale Lahden Vaaka/Lahti Precision Ltd., Lahti, Finland) and body condition score (BCS) [26] of the horses was monitored after each collection period.

### 2.2. Experimental Feeds and Feeding

The diets were formulated and adjusted to be as isocaloric as possible. The horses were individually fed at a level of 65–75 g DM kg ^−1^W^0.75^, corresponding to the feeding and energy level recommended in light work in accordance with the Finnish Feed Tables and Feeding Recommendations [19]. The regularly obtained BCS and BW were used to control their possible changes, and the individual energy intakes were adjusted if necessary. The horses were fed three times per day (at 6:00 a.m., 12:00 noon and 6:00 p.m.), except in the mornings of blood sampling days, when the forages were fed at 7:30 and concentrates at 8:00 o’clock.

The diets were (dry matter basis) (A) hay 100%; (B) haylage 100%; (C) hay 80% + whole oats 20%; (D) hay 65% + whole oats 35%; (E) hay 80% + commercial pelleted complete feed 20% (Lantmännen, Malmö, Sweden) and (F) hay 65% + commercial pelleted complete feed 35%. All diets except those including the complete feed were balanced with a mineral mixture (Ca:P = 3.57) in which P was in the form of monocalcium phosphate (Vilomix Ltd., Paimio, Finland) according to the P and Ca needs of the horses. The complete feed (the added P was in the form of monocalcium phosphate, Ca:P 1.43) covered the mineral requirements of the horses. Forages were fed from special hay-boxes to avoid dropping, and the concentrates were fed from feed mangers. Free water was available from float valve drinkers.

The dried hay was produced by a local farmer in Ypäjä (60°48′34′′ N, 23°16′35′′ E). The haylage (Prohay Ltd., Punkalaidun, Finland, 61°06′40′′ N, 23°06′20′′ E) was packed in 20 kg air tight plastic packages and purchased from the producer. The oats were produced by Luke.

Feed samples were collected daily and stored at –20 °C until analysis. The samples were analyzed at the Luke Laboratories (Jokioinen, Finland) for dry matter (DM), crude protein (CP), NDF, ADF, crude fiber (CF), and ash with standard wet chemical methods e.g., [27], as well as for P and Ca content using the method by Huang and Schulte [28]. The chemical composition of the feeds is presented in Table 1.

### 2.3. Feces Sampling and Analysis

Feces were collected for four days (Monday–Friday). Before the start of the collection period, the peat bedding was removed from the boxes and replaced with rubber mats. The feces collected overnight (every day at 9:00 a.m.) was thoroughly mixed and weighed, and a representative sample was taken. Partial samples were pooled throughout the collection period and stored at −20 °C. The daily amount of sampled feces was 12% of the total daily amount collected. Feces that were entangled with foreign substances such as urine were defined as waste. The amount of waste feces was weighed but not utilized in the analysis.

Fecal samples were analyzed at the Luke Laboratories for dry matter (DM), nitrogen, NDF, ADF, CF ash as well as total and (water) soluble P content. Nitrogen was analyzed with the Khjeldal method (AOAC-984.13) using Foss Khjeltec 2400 analyzer (Foss Analytical AB, Höganäs, Sweden), and the CP content was calculated as 6.25 × N. NDF and ADF were analyzed with ANKOM 220 fiber analyzer (ANKOM Technology, Macedon, NY, USA) using 25 µm nylon bags [29,30]. Total P was analyzed spectrometrically (ICP-OES, Thermo Jarrel Ash Iris advantage, Franklin, MA, USA). The proportion of soluble P (phospahte-P, PO_4_-P) was analyzed from 1:60 water extracts using a continuous photometric flow analyzer (Aquakem 250, Thermo Fisher Scientific Inc., Vantaa, Finland) as described by Keskinen et al. [31]. Because only a very low proportion (about 1%) of the phosphorus is excreted in the urine [8,10,12], urine was not collected in this experiment.

### 2.4. Blood Samples and Analysis

Blood samples were collected 90 min after the morning meal every Wednesday during the collection period. A blood sample was drawn from the jugular vein into two sample tubes (2 × 10 mL). The samples were analyzed for inorganic P and Ca photometrically using wavelengths 660 nm and 340 nm, respectively. The analyses were performed in a clinical authorized laboratory (Ellab Ltd., Ypäjä, Finland).

### 2.5. Statistical Analysis

Differences in digestibility, as well as intake, excretion and retention parameters between the diets were statistically analyzed using the SAS (SAS 9.3, 2008) GLM procedure (SAS Institue, Cary, NC, USA) applying the following statistical model:

Y_ijk_ = µ_ijk_ + a_i_ + p_j_ + d_k_ + e_ijk_ (1)

where µ_ijk_ is the overall mean, a_i_ is the random effect of the animal (i = 1…6), p_j_ is the fixed effect of the period (j = 1…4), d_k_ is the fixed effect of the diet (k = 1…6), and e_ijk_ is the normally distributed error with a mean of 0 and variance δ^2^. The differences between the diets were tested with orthogonal contrasts: (1) B vs. A and C-F; (2) A vs. C-F; (3) C and D vs. E and F; (4) C vs. E and D vs. F; and (5) the interactions between the type of concentrate and concentrate level C and D vs. E and F, C vs. E, and D vs. F. Concerning the retention values, it was also tested if they differ from zero.

Differences in the proportion of soluble P of the excreted P were not studied because the diets were composed of different ingredients containing various sources of P (inorganic and organic sources, phytate P) that were not analyzed.

## 3. Results

### 3.1. Feed and Nutrient Intakes

The feed, energy and nutrient intakes for each diet are presented in Table 2. The DM intake was smallest in horses eating only haylage (*p* < 0.001), but same time they had the largest CP intake (*p* < 0.001). Concerning the concentrate supplemented diets (hay + oats or complete feed), the concentrate level did not affect the DM intake. The horses maintained their BW and BCS during the experiment (mean initial BW = 552 ± 32 kg, mean final BW = 558 ± 32 kg).

### 3.2. Intake, Fecal Excretion and Digestibility of Phosphorus

The average P intake was 22.0 ± 2.0 g/d. The P intake increased with an increasing concentrate intake (Table 3). Horses ingesting oats had larger daily intake of P (22.8–24.8 g) compared to those fed with the complete feed (20.9–24.1 g) (*p* < 0.001) and horses fed with hay only had smaller intakes than horses supplemented with concentrates (20.6 vs. 20.9–24.8 g) (*p* < 0.001). Horses fed with haylage had somewhat smaller P intake than those who ate hay (*p* = 0.036).

The average daily quantity of dung was 15.6 ± 2.5 kg/horse. The horses excreted an average of 20.9 ± 1.4 g/d P in feces. Excretion was smallest (20.0 g) in horses on a hay-only diet (*p* = 0.021) (Table 3). Horses supplemented with oats excreted somewhat more P (21.5–22.1 vs. 19.9–21.5 g) than those supplemented with the complete feed (*p* < 0.025), and the excretion increased with increasing concentrate intake (*p* = 0.033).

The horses were on a positive P balance in all diets (Table 3). The retention of P was largest in the diet D (with the highest complete feed level) being 2.8 g/d. The retention values were different from zero for the diets B (*p* = 0.002), C (*p* = 0.05), D (*p* <0.001), E (*p* = 0.08), and F (*p* = 0.002). The P retention increased (*p* = 0.0145) with the increasing concentrate level. Feeding concentrates slightly improved P digestibility (*p* = 0.024). The amount of water-soluble phosphorus of the P excreted in feces was 18.3 ± 2.5 g/d, on average. This corresponds to 87.6% of the P in feces.

### 3.3. Intake, Fecal Excretion and Digestibility of Calcium and Magnesium

The intake of calcium was largest in the horses fed with haylage only (*p* > 0.001) (Table 4). Ingestion of concentrates decreased Ca intake, depending on type of concentrate fed. Mg intake was smallest in the horses on a haylage-only diet. The intake increased when concentrates were fed. The increase was largest with complete feed (interaction *p* < 0.001).

Concerning the excretion of minerals, horses on the haylage-only diet excreted somewhat less Mg than fed with hay only (*p* = 0.03). Comparing the concentrates, horses supplemented with complete feed excreted more both Ca and Mg than those supplemented with oats (*p* = 0.048 and *p* < 0.001, respectively). The digestibility of Ca did not differ between the diets. Concerning Mg, digestibility was lowest in the haylage-only diet, and lower when oats was fed compared with feeding the complete feed. The variation in Mg digestibility values was large (Table 4).

### 3.4. Digestibility of the Diet Nutrients

The dry matter digestibility of the haylage-only diet was lower compared with the other diets (*p* < 0.001) (Table 5). Supplementing the forage diets with concentrates improved the digestibilities of crude protein (*p* = 0.002) and organic matter (*p* = 0.077) but the concentrate level fed did not affect the digestibility of the fiber fractions. The CP digestibility of the haylage-only diet was better (*p* = 0.009) compared to the other diets. Correspondingly, the CP digestibility of the hay-only diet was the lowest (*p* = 0.004).

### 3.5. Blood Concentrates of P and Ca

The between diet variation of the blood serum P and Ca concentrations was small. The average blood serum P concentration was 1.16 ± 0.04 mmol/L. Comparing concentrate types, the concentration was larger when oats was fed than when the complete feed was fed (1.21 vs. 1.13 mmol/L) (*p* < 0.031). The mean blood Ca concentration of the horses was 3.14 ± 0.04 mmol/L. It was larger when the horses were on a hay-only diet compared with the diets containing concentrates (3.22 vs. 3.12 mmol/L) (*p* < 0.001).

## 4. Discussion

The main goal of this experiment was to find the differences between the diets for P utilization, rather than the actual values for specific diets. Because studying digestibilities of other nutrients was only a secondary aim of this study, the results are discussed only briefly.

### 4.1. Feed Values and Nutrient Intakes

The feed values corresponded to the analyzed values presented for Finnish forages produced for horses [32], the hay being of “medium nutritional quality” and the haylage of “high nutritional quality”. Concerning oats, the CP content was lower than that presented in the Finnish Feed Tables and Feeding Recommendations (10.4. vs. 12–13%) [19]. The NDF, ADF, and CF values were also lower than the values presented for average Finnish oats [19]. The P, Ca, and Mg content of the forages and oats was lower than the values presented for hays and haylages [19]. The mineral content was also clearly lower than reported for Norwegian and Swedish haylage samples collected from horse farms [20].

The nutrient intakes naturally varied because of the differences in the composition of the feeds and actual intakes, although the individual diets were initially formulated and balanced to correspond the needs of each horse [19] and be as isocaloric as possible. The smaller DM intake of the horses in the haylage-only diet resulted from restriction of haylage intake because of its high CP content. This led naturally also to smaller energy (ME) intake. In addition, the size of the horse affected the individual daily portion such that larger horses had larger portions. The horses maintained their BW and BCS during the experiment, indicating that the feeds, feeding regime, and intakes applied covered the nutritional requirements of the horses (energy and protein) in the course of the experimental period The mean daily energy intake of 77.5 MJ ME/d during the course of the experiment agreed with the recommendation for horses in light work [19].

### 4.2. Intake, Fecal Excretion and Digestibility of Phosphorus

The differences in the P intake between the diets were due to the differences in the feeds and diet compositions, but the forms of P were not analyzed. P concentrations in oats and complete feed were higher than in the forages. In addition, the oat supplemented diets were balanced for minerals (P and Ca) with a mineral supplement mixture which also increased the P intake. The intakes of P were in accordance with the current recommendations [1,19], except on the highest concentrate levels where they exceeded the recommendations.

The excretion of phosphorus observed here was within the ranges presented in the literature [7,12,18,33,34]. P excretion is linearly related to its intake, and the intake increases with the increasing concentrate ingestion [10,12,33]. Van Doorn et al. [35] concluded that horses can regulate P digesting and thus P balance. The extra P can be excreted in the feces.

In the present study, the digestibility of P in adult horses varied from 2.7 to 11.1%, improving with an increased concentrate intake. This is well in line with the results of van Doorn et al. [18] for adult horses (2.4–15.4%). However, higher values (4.2–28.7%) have also been reported [15,34,36] for adult horses (with a range between 4 and 25 years). In many studies the digestibilities show impaired values with increasing age. The largest digestibilities (37–42%) have been reported for young horses (8-months-olds), but they decline quickly (to 2.0–7.7%) when the horses are between one and two years old [7,12]. Elzinga et al. [36] reported digestibility of 4.2% for aged (19–28 years) horses. P digestibility is therefore influenced by the age of the horse. In this study, the horses were between 5 and 13 years old, and P digestibility seems to accord well with previous studies when the age of the horses is considered. In previous studies, higher values have also been reported when hay + concentrates were fed e.g., [6,7,15,18,34,35] compared with forage-only diets [12,28]. The digestibility of P may also improve somewhat with increasing P intake [6]. Furthermore, digestibility is affected by the components (feeds) of the diet [1].

Diets with the highest concentrate levels (35% oats or complete feed) had better digestibility compared to the other diets. All diets resulted in a positive P balance and the P retention differed from zero in all except the only-hay diet, in which the intake was lower compared to the other diets. According to previous studies [10,18], the P retention increased with P intake in adult animals. The P retention values observed in our study were smaller (less than half) than reported for adult (appr. 6-year-olds) Standardbred horses [18] fed mixed hay + concentrate diets, but in the same time, the intakes were also correspondingly smaller. According to that study, the reasons for the P retention in adult horses are not known. However, Buchholz-Bryant [37] reported a higher P retention in mature horses (7 to 11 year old) at rest compared to exercised horses. In the present study the horses were only freely exercised daily in outdoor paddocks for 2–4 h, and during the collection period, they were walked manually in the stable corridors for 15 min In the study of Van Doorn et al. [18], the horses were given 1-h walk on a treadmill, and during the collection period they walked manually for 10 min indicating rather light work level. Thus, the light work load of the horses may be one reason for the P retention in our study. In addition, because phosphorus is stored in many body tissues and fluids [1,2,3], some P can be accumulated to these, too. The retention values presented here includes also the urine P, because urine was not collected and analyzed for P. This was done, because according to studies [8,10,12], only a very low proportion (about 1%) of the phosphorus is excreted in the urine.

The Ca:P ratio of the feed or diet affects not only the digestibility of phosphorus but also the digestibility of calcium [15]. A high calcium intake can impair phosphorus digestibility at a Ca:P ratio of 2.58 or more [1,18]. However, the dietary Ca:P ratios in the current study were much lower, 1.14–1.58. Concerning fecal Ca:P ratios, Böswald et al. [38] found that in horses (and other large hindgut fermenters), the fecal Ca:P ratio is lower than the dietary Ca:P ratio. The present study was in accordance with this, the mean fecal ratios ranging from 0.80 to 0.95.

### 4.3. Intake, Fecal Excretion and Digestibility of Calcium and Magnesium

Calcium and magnesium intakes also differed between the diets because of the differences in the mineral content of the feeds. The intakes of Ca and Mg agreed with the recommendations [1,19], with the exception that Mg intake was above the recommended values when complete feed was fed. Intakes of Ca from the concentrates were small. Oats was low in Ca, and the increased proportion of oats and decreased proportion of forage in the diet resulted in a decline in Ca intake. Concerning the intakes of Mg, the larger Mg content of the complete feed compared to that of oats explains the differences in Mg intake.

The excretion of Ca in the present study was smaller than reported in the literature e.g., [7,18] because the intake was smaller. It is also likely that different Ca sources have an influence. The Mg excretion agreed with previous studies [7,18].

The amounts of excreted Ca and Mg in feces are related to intakes [39,40], but no effect of intake was observed by Nielsen et al. [33]. Meyer et al. [41,42] reported that diets containing more roughage resulted in a higher renal excretion of Ca and Mg.

The observed digestibilities of Ca not differing between the diets agreed with the literature values [2,7,18,35]. Van Doorn et al. [18] have reported that high amounts of P and phytate P may decrease Ca digestibility. The larger excretion of Mg compared with its intake in all diets, except those including the complete feed, explains, the poor Mg digestibility in these diets. The digestibility of Mg has been reported to be largely varying, and negative values have also been reported [2,7,18,35], as in the present study. Because Ca and Mg are excreted in large quantities in urine [9,40], the observed digestibilities here may be underestimated.

### 4.4. Digestibility of the Diet Nutrients

It is very likely that the lower DM digestibility of the haylage-only diet was due to its larger fiber content (NDF, ADF, CF) compared to the other diets. The better CP digestibility of the haylage-only diet was due to its larger CP content, and correspondingly, the poor CP digestibility of the hay-only diet was due to its low CP content. These results are supported for example by Särkijärvi and Saastamoinen [27] and Ragnarsson and Lindberg [43,44]. The positive effect of including concentrates in the forage diets agrees with previous studies, being e.g., due to the lower NDF content of the diet e.g., [45,46]. The NDF content also explains the digestibility value differences between the forage diets. The digestibility values observed in the present study are comparable with those reported previously for Finnhorses of the same age e.g., [27,47].

### 4.5. Blood Concentrates of P and Ca

The blood phosphorus levels are affected by phosphorus intake [48,49], which may explain why blood levels were highest in the horses whose diets were supplemented with oats. Greiwe-Crandell et al. [50] suggested that mares fed an all-forage diet marginal or low in phosphorus may mobilize P from bone.

The blood Ca concentration was largest in the horses on the hay-only diet. Meyer et al. [42] reported higher plasma Ca levels for forage fed horses than concentrate fed horses. Some other studies, however, pointed out that the blood Ca concentration does not depend on the Ca intake [48,49]. Regarding all diets, the average blood serum Ca and P concentrations were within the normal ranges used for Finnhorses (https://www.movet.fi/laboratoriokasikirja/). To maintain physiologic Ca and P blood levels, mammals can absorb them from the gastrointestinal tract or change their bone turnover [38].

### 4.6. Impact of Horse Diets on P Leaching

The daily quantity of dung produced by the horses was in line with the literature values [7,51,52], depending, however, on diet and feed intake. The average daily phosphorus excretion of about 21 g in feces in this study, when typical diets and current recommendations [1,19] were applied, resulted in 7.6 kg P per year. If the P is not properly absorbed in stable beddings, or if the dung in paddocks is altered by rain and water from melting snow, P in feces and manure may present an environmental risk when leaching into waters.

The soluble P part of the total P in feces available for the utilization of plants accounted for about 88% of the P excreted in feces in this study. Ögren et al. [12] reported a proportion of 80% of soluble P. The P that is unavailable is vulnerable to runoff losses. According to Chapuis-Lardy et al. [53], excess dietary P is excreted in feces in water-soluble forms. Dougherty et al. [54] pointed out that around 90% of P losses occurred in water-soluble form. The leaching P from the dung of horses is mainly inorganic [55]. Consequently, the argument of the NRC [1] that horse manure is less harmful to the environment compared with that from other farm animals because of its low proportion of water-soluble P, is not correct in the light of the results of this and previous studies.

It is possible that the composition of the diet affects the solubility of P, as reported for dairy cows and pigs [56,57]. However, in this study it was irrelevant to compare the diets because of their composition, i.e., the inclusion of various P sources in the same diet. Further studies can be suggested to be carried out concerning this issue also in horses. Ögren et al. [12] concluded that soluble P has a strong positive relationship to P intake in horses.

As P loss is linearly related to its intake in various animal species [12,53,58], it is impossible to conclude how polluting horse industry is compared with other forms of animal production. However, in the study of Ögren et al. [12], the high proportion of inorganic P in horse feces indicated that P overfeeding of horses might be more harmful to the environment than P overfeeding of dairy cows. Previously, several other authors have also reported that horse paddocks may pose a high risk of extensive P loss [22,23,24,25,55,59]. Regular removal of dung from paddocks is recommended to minimize this risk [25,55,60,61]. How often this should be done naturally depends on the time the horses spend in the paddocks and livestock density/ha. Phosphorus sorbing materials (e.g., Fe containing) [22,59], filtering materials (geotextile-gravel) [61], or organic (bedding) materials [62] can also be used on paddock surfaces to reduce leaching loss.

Ögren et al. [12] concluded that an increase in the P requirement for growing horses is not justified. The present study shows that it is unnecessary to supplement the diets of mature horses, especially those in light work, with inorganic phosphorus, when the diets are supplemented with concentrates. According to Fowler et al. [7], the organic P in feeds may fulfill the needs of horses in light work, and no supplementation with inorganic P is needed. Balancing the diets for P intake can be estimated to save both money and environment in dairy production [63]. There may also be economic motives to catch the P in feces and absorb it in bedding materials, because the use of horse manure may reduce fertilizing costs. When horse manure is composted, its nutrients can be recycled and utilized [31], which reduces the use of inorganic fertilizers.

In addition, optimizing the proportions of the diet components, for example by supplementing the forage diets with concentrates, may improve the digestibility of phosphorus. However, it is necessary to analyze the feeds for the mineral concentrations because of the large variation [20,32,64]. In complete feeds for horses, P (and other mineral) concentrations are usually formulated to cover the requirements of an “average horse” when “medium-quality” forages are fed. They thus do not take into account the true mineral concentrations in the other components of the diet. This may result in over- or undernutrition in practical feeding. As in this study, they also contain the added P in inorganic form. In addition, when increasing the proportion of concentrates in the daily ration, the possible detrimental effects of starch [65] have to be considered. In the present study, the concentrate levels fed were not very large, and the diets were based on forages.

## 5. Conclusions

Horse dung may pose a potential risk of P leaching into the environment, and is not less harmful to the environment than that from other farm animals if not properly managed, because most of the P in feces is in soluble form. Supplementation with inorganic P should be controlled in the diets of adult horses in light work to decrease the excretion of P in feces. Supplementing forage diets with concentrates may improve the digestibility of phosphorus and, thus, improves the availability of P to horses. More research especially into cost effective feeding strategies and their applications for horses is essential, e.g., concerning diet composition and ingredients, to reduce horse industry’s harmful impacts on and risks to water quality.

## Figures and Tables

**Table 1:** Average chemical composition (g/kg DM) and energy value (ME MJ/kg DM) of the experimental feeds.

Composition	Hay	Haylage	Oats	Complete Feed	Mineral Mixture ^1^
Dry matter	83.6	59.4	86.1	86.9	96.0
Crude protein	82.1	122.5	107.0	124.4	
Crude fiber	318.7	326.7	103.9	113.5	
NDF	596.0	615.0	260.5	305.4	
ADF	317.5	326.6	104.3	123.3	
Ash	62.3	67.6	32.2	74.0	737.8
Calcium	2.4	2.9	0.5	7.0	156.4
Magnesium	1.2	1.3	1.3	4.2	45.0
Phosphorus	2.2	2.7	3.6	4.9	43.6
Energy ME	9.7	9.6	12.0	11.0	

NDF = neutral detergent fiber; ADF = acid detergent fiber; ^1^ Inorganic mineral sources: monocalcium phosphate, limestone meal, magnesium oxide; ME = metabolisable energy.

**Table 2:** Mean daily energy (MJ ME), dry matter (g) and nutrient intakes (g) for the experimental diets.

Diet	A	B	C	D	E	F	Pooled SEM	Statistical Significance (*p*-Values)				
Forage	Hay	Haylage	Hay	Hay	Hay	Hay		Haylage vs. Others	Hay vs. ConS	Oats vs. Comp	ConL	ConT × ConL
ConL	0	0	O20	035	C20	C35						
ME	74.3	73.3	79.1	82.6	77.6	78.7	0.4	<0.001	<0.001	<0.001	<0.001	0.009
DM	7536	6702	7568	7734	7601	7595	17.0	<0.001	0.038	0.037	0.439	0.338
OM	6981	6186	7148	7250	7081	7075	47.3	<0.001	0.033	0.027	0.368	0.272
CP	607	812	647	705	685	744	17.3	<0.001	0.014	0.047	0.009	0.979
CF	2366	2155	2079	1852	2102	1873	16.4	<0.001	0.001	0.196	<0.001	0.946
NDF	4427	4060	3992	3647	4073	3753	33.9	0.055	<0.001	0.018	<0.001	0.715
ADF	2361	2155	2076	1845	2109	1891	18.8	<0.001	<0.001	0.058	<0.001	0.746
Ash	556	516	510	485	519	521	17.0	0.901	0.033	0.202	0.519	0.446

ME = metabolisable energy MJ/day; DM = dry matter; OM = organic matter; CP = crude protein; CF = crude fiber; NDF = neutral detergent fiber; ADF = acid detergent fiber; O = Oats; C = Complete feed; ConL = Concentrate level (20 or 35% of oats O or complete feed C); ConS = Concentrate supplementation; ConT = concentrate type (oats/complete feed); Comp = complete feed.

**Table 3:** Daily intake (g), fecal excretion (g), digestibility (%) and retention (g) of phosphorus (P).

Diet/Forage	A	B	C	D	E	F	Pooled SEM	Statistical Significance (*p*-Values)				
	Hay	Haylage	Hay	Hay	Hay	Hay		Haylage vs. Others	Hay vs. ConS	Oats vs. Comp	ConL	ConT × ConL
ConL	0	0	O20	O35	C20	C35						
Intake P	32.6	34.2	29.6	28.3	24.7	30.4	0.45	<0.001	<0.001	0.010	0.001	<0.001
Excretion P	19.1	17.7	17.5	17.8	19.4	20.2	0.98	0.330	0.757	0.048	0.610	0.797
Digestibility P	41.6	47.1	29.3	38.1	22.6	31.7	7.10	0.096	0.187	0.371	0.265	0.987
Retention	0.6	1.9	1.0	2.8	0.9	1.9	0.45	0.354	0.075	0.25	0.014	0.379

O = Oats; C = Complete feed; ConL = Concentrate level (20 or 35% of oats O or complete feed C); ConS = Concentrate supplementation; ConT = concentrate type (oats/complete feed); Comp = complete feed.

**Table 4:** Daily intake (g), excretion (g) and digestibility (%) of calcium (Ca) and magnesium (Mg).

Diet/Forage	A	B	C	D	E	F	Pooled SEM	Statistical Significance (*p*-Values)				
	Hay	Haylage	Hay	Hay	Hay	Hay		Haylage vs. Others	Hay vs. ConS	Oats vs. Comp	ConL	ConT × ConL
ConL	0	0	O20	O35	C20	C35						
Intake												
Ca	32.6	34.2	29.6	28.3	24.7	30.4	0.45	<0.001	<0.001	0.010	0.001	<0.001
Mg	9.31	8.71	9.25	9.74	13.9	17.3	0.26	<0.001	<0.001	<0.001	<0.001	<0.001
Excretion												
Ca	19.1	17.7	17.5	17.8	19.4	20.2	0.98	0.330	0.757	0.048	0.610	0.797
Mg	10.4	10.1	10.1	10.4	10.6	12.9	0.28	0.030	0.107	<0.001	0.002	0.006
Digestibility												
Ca	41.6	47.1	29.3	38.1	22.6	31.7	7.10	0.096	0.187	0.371	0.265	0.987
Mg	−13.1	−19.0	-9.0	-7.0	23.4	25.3	4.73	0.001	0.002	<0.001	0.702	0.993

O = Oats; C = Complete feed; ConL = Concentrate level (20 or 35% of oats O or complete feed C); ConS = Concentrate supplementation; ConT = concentrate type (oats/complete feed); Comp = complete feed.

**Table 5:** Apparent digestibility coefficients (%) of the diet nutrients.

Diet/Forage	A	B	C	D	E	F	Pooled SEM	Statistical Significance (*p*-Values)				
	Hay	Haylage	Hay	Hay	Hay	Hay		Haylage vs. Others	Hay vs. ConS	Oats vs. Comp	ConL	ConT × ConL
ConL	0	0	O20	O35	C20	C35						
DM	55.0	49.9	56.6	59.3	57.5	60.1	1.33	<0.001	0.046	0.543	0.090	0.998
Ash	38.4	31.6	29.8	29.6	34.9	37.7	2.73	0.437	0.105	0.033	0.666	0.591
OM	56.3	51.4	58.5	61.3	59.1	61.8	1.29	<0.001	0.023	0.699	0.077	0.972
CP	50.9	63.6	51.7	61.8	57.3	63.6	1.83	0.009	0.004	0.066	0.002	0.310
CF	49.5	45.7	46.1	43.0	49.6	56.5	2.61	0.685	0.291	0.206	0.294	0.997
NDF	49.5	46.3	46.3	42.5	49.0	47.5	2.44	0.811	0.278	0.144	0.334	0.635
ADF	47.2	41.6	41.7	41.4	45.5	40.5	3.40	0.668	0.224	0.669	0.488	0.497

DM = dry matter; OM = organic matter; CP = crude protein; CF = crude fiber; NDF = neutral detergent fiber; ADF = acid detergent fiber. O = Oats; C = Complete feed; ConL = Concentrate level (%); ConS = Concentrate supplementation; ConT = concentrate type (oats/complete feed); Comp = complete feed.
