# Erratum: Saastamoinen, M.; Särkijärvi, S.; Valtonen, E. The Effect of Diet Composition on the Digestibility and Fecal Excretion of Phosphorus in Horses: A Potential Risk of P Leaching? *Animals* 2020, *10,* 140

**DOI:** 10.3390/ani10020285

**Published:** 2020-02-12

**Authors:** Markku Saastamoinen, Susanna Särkijärvi, Elisa Valtonen

**Affiliations:** 1 Natural Resources Institute Finland, FI-00790 Helsinki, Finland; susanna.sarkijarvi@luke.fi; 2 Department of Animal Science, University of Helsinki, FI-00790 Helsinki, Finland; elisa.mj.valtonen@gmail.com

The authors wish to make the following corrections to their paper [1]:

In Table 3, the rows showing “Intake P”, “Excretion P” and “Digestibility P” have typing errors and are now corrected (see the corrected version of Table 3 below).

## Figures and Tables

**Table 3:** Daily intake (g), fecal excretion (g), digestibility (%) and retention (g) of phosphorus (P).

Diet/Forage	A	B	C	D	E	F	Pooled SEM	Statistical Significance (*p*-Values)				
	Hay	Haylage	Hay	Hay	Hay	Hay		Haylage vs. Others	Hay vs.ConS	Oats vs.Comp	ConL	ConT × ConL
ConL	0	0	O20	O35	C20	C35						
Intake P	20.6	22.1	22.8	24.8	20.9	24.1	0.21	0.036	<0.001	<0.001	<0.001	0.027
Excretion P	20.0	20.2	21.5	22.1	19.9	21.5	0.42	0.125	0.021	0.025	0.033	0.251
Digestibility P	2.7	8.0	5.6	11.1	4.9	10.6	1.98	0.652	0.037	0.761	0.024	0.974
Retention	0.6	1.9	1.0	2.8	0.9	1.9	0.45	0.354	0.075	0.25	0.014	0.379

O = oats; C = complete feed; ConL = concentrate level (20 or 35% of oats O or complete feed C); ConS = concentrate supplementation; ConT = concentrate type (oats/complete feed); Comp = complete feed.

## References

[1] Saastamoinen M., Särkijärvi S., Valtonen E. (2020). The Effect of Diet Composition on the Digestibility and Fecal Excretion of Phosphorus in Horses: A Potential Risk of P Leaching?. Animals.

